# Overlapping imaging features between miscarriage of a low‐lying gestational sac and cervical ectopic pregnancy

**DOI:** 10.1002/ajum.12337

**Published:** 2023-02-27

**Authors:** Jessica Teoh, Sumathi Rajendran, Sarika Gupta

**Affiliations:** ^1^ Women and Babies Department Royal Prince Alfred Hospital Camperdown New South Wales Australia; ^2^ Sydney Ultrasound for Women Sydney New South Wales Australia

**Keywords:** cervical ectopic pregnancy, haemorrhage, low‐lying gestation sac, miscarriage

## Abstract

Early pregnancy ultrasound must satisfy objective criteria to make a safe diagnosis of miscarriage. The differential diagnosis of low‐lying gestational sac includes cervical stage of miscarriage and cervical and caesarean scar ectopic pregnancies. Misdiagnosis can lead to significant maternal morbidity. We describe a pregnancy in a 36‐year‐old primiparous woman where ultrasound findings of a low‐lying gestation sac satisfied criteria for miscarriage; however, dilatation and curettage of pregnancy contents resulted in brisk cervical bleeding. Ultrasound at 6 weeks 6 days of gestation showed an intra‐uterine pregnancy of uncertain viability. Repeat scan after 11 days confirmed miscarriage based on an absence of interval progression between scans and no embryonic heartbeat. The collapsed gestational sac (GS) was seen at the level of the internal os with decidual reaction and peri‐trophoblastic blood flow. Inferior to the sac, minimally vascular trophoblastic appearing tissue was beginning to distend the upper cervical canal: the sliding sign was positive for the GS and negative for the upper cervical contents. Cervical stroma was clearly seen circumferential to the distending tissue. The patient underwent dilatation and curettage of the uterus complicated by 2000 ml haemorrhage requiring blood transfusion and medical and surgical management with intra‐cavitary placement of a Foley catheter. Histopathology confirmed pregnancy tissue with the disruption of cervical epithelium but no true invasion. The patient was counselled to attend a specialist obstetric imaging facility for an early dating ultrasound in future pregnancies. The current body of literature does not describe cases of low‐lying gestation sac miscarriage with high‐risk features of trophoblastic extension into the cervical canal. We suggest maintaining a high index of suspicion and excluding differential diagnoses as the majority of women have no risk factors for ectopic pregnancy. These cases should be recommended for surgical management.

## Background

Early pregnancy ultrasound must satisfy criteria for a safe diagnosis of miscarriage.^1^ The differential diagnosis of low‐lying gestational sac includes cervical stage of miscarriage, morbidly adherent placenta, cervical or caesarean scar ectopic pregnancy.^2^ Although cervical ectopic pregnancies account for <1% of all ectopic pregnancies, their incidence is increasing.^3^ Misdiagnosis can lead to significant maternal morbidity. We describe a case where ultrasound features of miscarriage are satisfied; however, some of the features overlap with cervical ectopic pregnancy.

## Case report

We describe a case of spontaneous pregnancy in a 36‐year‐old primiparous woman in which ultrasound findings of a low‐lying gestation sac satisfied criteria for miscarriage; however, dilatation and curettage of products resulted in brisk cervical bleeding.

**Figure 1:**
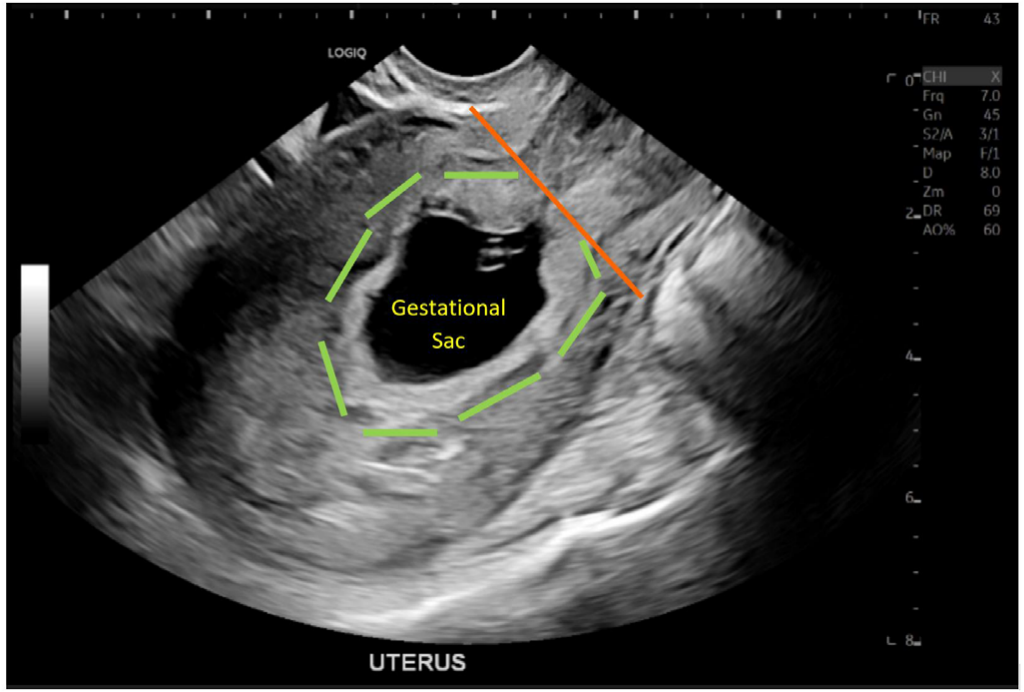
Outpatient obstetric ultrasound shows mid‐sagittal view of the uterus at 6 weeks 6 days of gestation (based on last menstrual period). Gestational sac lies in a low position within the uterus (green outline) with irregular contour above the level of the internal os (orange line). There is a yolk sac and a fetal pole. Crown‐rump length measures 8.3 mm, yolk sac 1.8 mm, and fetal heart rate was low at 74 bpm. This was originally reported as intra‐uterine pregnancy of uncertain viability with poor prognostic factors of fetal bradycardia, low‐position and irregular gestational sac outline.

**Figure 2:**
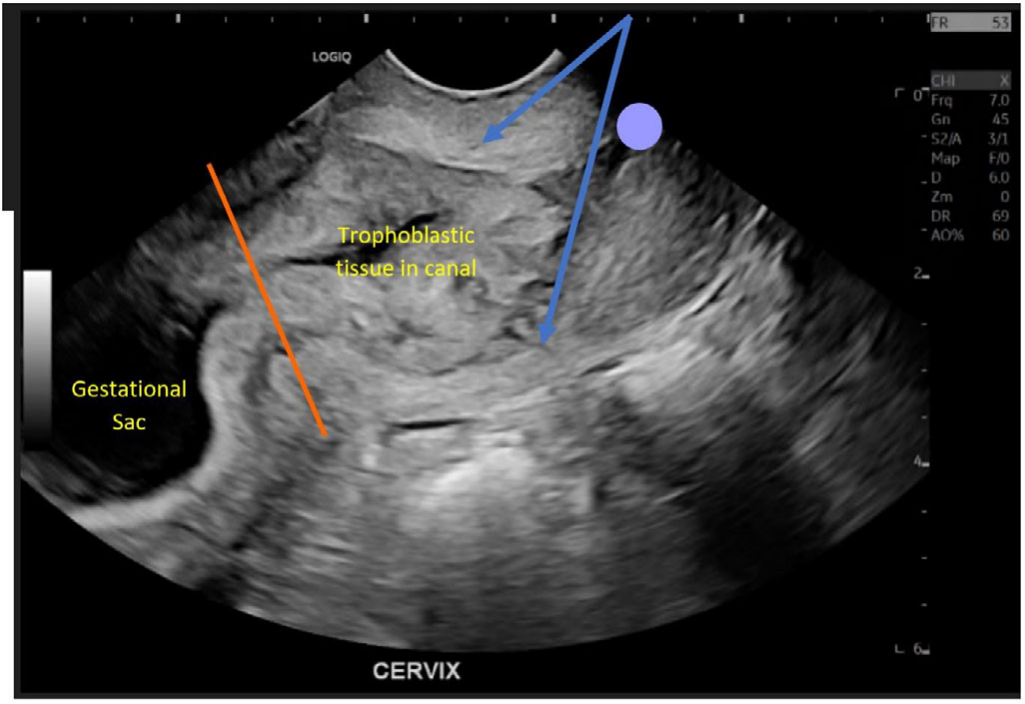
Outpatient ultrasound at 6 weeks 6 days of gestation demonstrates sagittal view of the lower uterine segment and the upper cervical canal (orange line marks the internal cervical os). The cervical canal contains echogenic tissue continuous with the gestational sac. Cervical stroma can be seen around the echogenic tissue (blue arrows), and the external cervical os appears closed (purple dot). Extension of vascular echogenic tissue into the cervical canal that is continuous with the gestational sac should be considered a red flag feature to prompt further investigation to exclude differentials such as cervical ectopic pregnancy. This finding was not commented upon in the report, nor were any colour images of the endocervical canal contents supplied.

**Figure 3:**
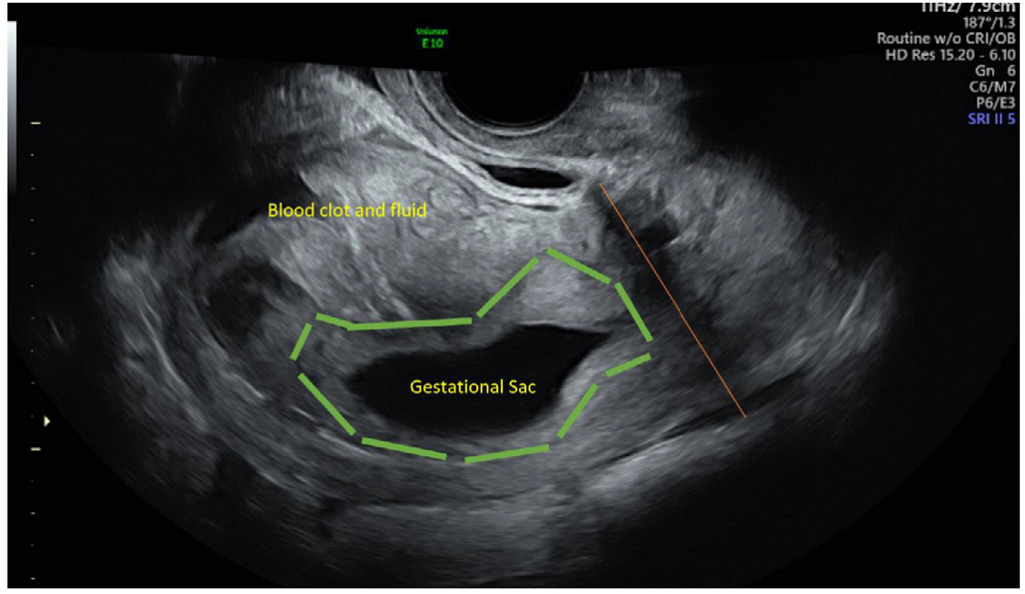
Mid‐sagittal view of the uterus at 8 weeks 3 days of gestation (gestation based on initial dating ultrasound). The upper cavity is distended by heterogeneous material suggestive of intracavitary fluid and blood. The elongated and irregular outline of the gestational sac can be seen in the lower cavity, above the level of the internal os with a surrounding decidual reaction (outlined in green). There is no significant pelvic free fluid. The orange line indicates the level of the internal os.

**Figure 4:**
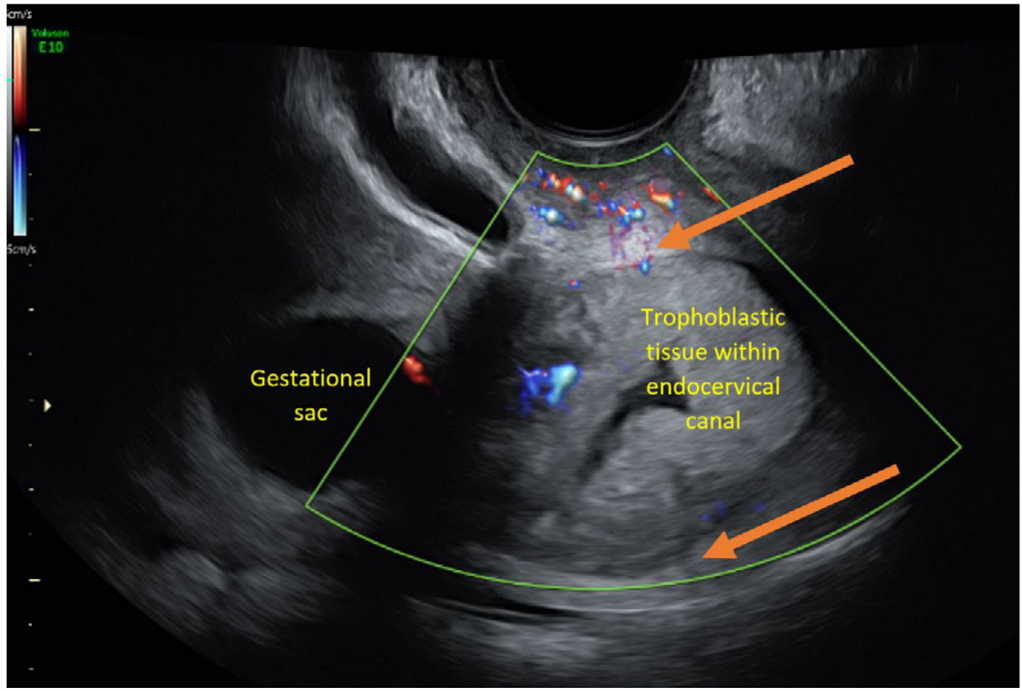
Mid‐sagittal view (at 8 weeks 3 days of gestation) of the lower uterine segment and upper cervical canal demonstrating distention by trophoblastic tissue. The cervical stromal margins (orange arrows) are visible peripherally, indicating the tissue is within the cervical canal and not intercepting into the cervical stroma. The gestational sac can be seen above the level of the internal os with minimal vascularity between the sac and the extending trophoblastic tissue.

An outpatient ultrasound made at 6 weeks 6 days of gestation reported an intra‐uterine pregnancy of uncertain viability with fetal bradycardia and crown‐rump length (CRL) of 8.3 mm, and mean sac diameter (MSD) of 31 mm (Figure 1). The images also showed an irregular gestational sac with fetal heart activity at 74 bpm sitting low in the cavity above the level of the internal os. Echogenic tissue could be seen extending into the cervical canal; however, this was not flagged as a suspicious finding (Figure 2). Based on poor prognostic features (low fetal heart rate, and irregularity and position of the sac), repeat beta‐hCG test and ultrasound were recommended in 10 days. Repeat tertiary ultrasound 11 days later demonstrated similar findings (the irregular gestational sac above the level of the internal os with a double‐decidual ring sign and a small amount of peri‐trophoblastic blood flow). There was no fetal heart activity (CRL 3.3 mm), and the upper cavity was distended by a large extramembranous haemorrhage (30 mL) with evidence of fresh clot (Figure 3, Video S1). The sliding sign was noted to be atypical being positive for the gestational sac above the internal os and negative for the trophoblastic tissue in the cervical canal. Cervical stroma could be appreciated circumferentially around the canal contents (Figure 4).

The patient elected for surgical management (dilatation and curettage of the uterus). Vaginal examination prior to the dilatation and curettage showed an anteverted uterus with cervix 1 cm dilated. The cervix was easily dilated to Hegar 8. Suction curettage was performed until the cavity clinically felt empty; however, haemorrhage was reported from the internal lip of the cervix. The vagina was packed and bimanual compression was applied. Medical management was instituted simultaneously with intravenous oxytocin, intravenous and intramuscular ergometrine and tranexamic acid. Haemostasis was achieved following the intracavitary placement of a Foley catheter inflated to 40 mL. Estimated blood loss was 2000 mL, and haemoglobin intraoperatively was 78 (from 101). She was administered 4 L of crystalloid and 1 unit of packed red blood cell transfusion. The Foley catheter was deflated in stages and removed on Day 1. The woman received an iron infusion prior to discharge on Day 2.

Histopathology showed disruption of cervical epithelium without true invasion. She was commenced on progynova and primolut to reduce the risk of postsurgical adhesions. The patient later followed up with her gynaecologist and was counselled to attend a specialist obstetric imaging facility for an early dating ultrasound in the next pregnancy.

## Discussion

In current practice, the diagnosis between cervical stage of miscarriage and ectopic pregnancies in the cervical and lower uterine segment is made based almost entirely on ultrasound criteria rather than histopathology.^1^ The diagnosis of miscarriage relies on conservative ultrasound criteria that result in 100% specificity.^1^ Despite having poor prognostic indicators at the time of initial imaging, including fetal bradycardia, irregular gestational sac outline and intrauterine hematoma, these are not recognised as initial or follow‐up criteria for diagnosing miscarriage.^1^ Miscarriage was diagnosed based on the previously documented loss of fetal heart activity and nonprogression of CRL.^1^

The current body of literature does not describe cases of low‐lying gestation sac miscarriage with high‐risk features of trophoblastic extension into the cervical canal. The patient's ultrasound findings were inconsistent with the diagnostic criteria for cervical ectopic pregnancy, which include the following: an empty uterus, a gestational sac positioned below the level of the internal cervical os, a barrel‐shaped cervix, a negative ‘sliding sign’ and peri‐gestational sac vascularity on colour Doppler.^4^ However, a previous study has described an ultrasound‐based category ‘low‐lying implantation ectopic pregnancy (LLIEP)’ representing a cluster of diagnoses including cervico‐isthmic pregnancies, cervical ectopics and caesarean scar ectopics, which are associated with overlapping clinical characteristics, response to treatment and outcomes.^5^ The patient did not have any ectopic pregnancy risk factors and most likely had a spontaneous pregnancy which implanted very low in the uterine cavity, and then had trophoblastic extension rather than invasion into the cervical canal. This sequence may explain the atypical sliding sign in which the pregnancy contents above the internal os appeared mobile relative to the inferiorly extending trophoblastic tissue; typically, the sliding sign appears negative in a cervical ectopic due to the closed internal os and the absence of tissue in the endo‐cervical canal.^4^ Interestingly, up to 50% of ectopic pregnancies occur in women without risk factors. Known risk factors for ectopic pregnancy include *in vitro* fertilisation, pelvic inflammatory disease, intrauterine contraceptive devices, caesarean section, prior cervical procedures and structural uterine anomalies.^6^

Currently, there is no consensus on the optimal management of miscarriage in low‐implanted pregnancies. Clinical management is aimed at minimising morbidity and preserving fertility.^4^ The patient in this case elected for surgical management. Pregnancies that implant near the cervix are at a significantly higher risk of haemorrhage: the thin cervical mucosa allows proliferating chorionic villi to deeply penetrate the underlying fibromuscular layer that has a rich blood supply from the uterine arteries.^7^ Therefore, it is important to delineate this entity apart from those in the cervical phase of miscarriage, especially as microscopic stromal invasion cannot be appreciated on imaging.^7^ Haemorrhage should be anticipated and surgery performed in an appropriate facility with active management, including uterotonics, cervical tamponade with a Foley catheter, cervical cerclage, vaginal ligation of cervical arteries or angiographic embolisation of the cervical, uterine or internal iliac arteries.^4^

According to previous case series, management of low implanted pregnancies is rarely expectant or medical due to the higher risk of failure and bleeding.^5^ In these case series, surgical management with concurrent tamponade was associated with high success rates and low complication rates.^5^ Medical management has also been reported in the context of haemodynamically stable patients with nontubal ectopics; however, its role in the management of low‐implanted pregnancies remains unclear.^8^ Similarly, expectant management for low‐implanted pregnancies has not been described in the literature.

## Conclusion

This case study highlights the clinical importance of correctly characterising a low‐implanted gestational sac that has overlapping ultrasound features with cervical ectopic pregnancy. We suggest maintaining a high index of suspicion for ectopic pregnancy in all early pregnancy ultrasounds with atypical features, especially those with a combination of low‐lying gestational sac, irregular sac outline and extension of echogenic tissue in the cervical canal, and to err on the side of caution with management counselling.

## Authorship statement

Jessica Teoh was involved in writing original draft, review and editing. Sumathi Rajendra was the supervisor and involved in conceptualisation of case report.Sarika Gupta was also involved in conceptualisation, writing, editing and reviewing the final article.

## Conflict of interest

The authors declare no competing interests.

## Funding

No funding information is provided.

## Consent statement

Signed consent for publication of this case has been obtained from the patient.

## Ethical approval

This case report was reviewed (Ethics Review Committee [RPAH Zone] Meeting, 1 July 2022. HERC\EXECOR\22‐07) and no ethical concerns regarding the case report and the publication of results in peer‐reviewed journals were raised.

## Supporting information


**Video S1.** Sagittal sweep of the uterus (at 8 weeks 3 days of gestation) demonstrating heterogeneous clot in the upper cavity, a lowly implanted gestational sac containing early fetal parts with a decidual reaction. The sac lies above the level of the internal os and trophoblastic tissue can be seen extending into the upper cervical canal.
